# Supplementary material for: The long-term impact of the Covid-19 pandemic on financial insecurity in vulnerable families: Findings from the Born in Bradford Covid-19 longitudinal study
Source: PLoS One. 2023 Nov 29;18(11):e0295064. doi: 10.1371/journal.pone.0295064 (PMC10686492; doi:10.1371/journal.pone.0295064)
Supplement: Attachment — Submitted filename: Response to Reviewers.docx [file pone.0295064.s001.docx]

Sian Reece

Hull York Medical School

Heslington Road

York

YO10 5DD

Friday 26^th^ May 2023

Dear Wajiha Haq,

**Re: Manuscript PONE-D-23-05276 Revision Required**

Thank you for taking the time to consider our manuscript entitled “The long-term impact of the Covid-19 pandemic on financial insecurity in vulnerable families: findings from the Born in Bradford Covid-19 longitudinal study.”

We have carefully worked through the thoughtful comments of the editorial and reviewer teams and have provided our response to these comments for your consideration below.

1. Please ensure that your manuscript meets PLOS ONE's style requirements, including those for file naming. The PLOS ONE style templates can be found at 
<https://journals.plos.org/plosone/s/file?id=wjVg/PLOSOne_formatting_sample_main_body.pdf> and 
<https://journals.plos.org/plosone/s/file?id=ba62/PLOSOne_formatting_sample_title_authors_affiliations.pdf>

Thank you for the feedback and for signposting us to the relevant formatting guidance. We have now amended the manuscript and relevant files as per the PLOS ONE style templates.

2. Please ensure that you have specified (1) whether consent was informed and (2) what type you obtained (for instance, written or verbal, and if verbal, how it was documented and witnessed). If your study included minors, state whether you obtained consent from parents or guardians. If the need for consent was waived by the ethics committee, please include this information.

We have specified this information under the ‘Ethics’ heading found within the ‘Methods’ section of the manuscript.

3. We note that the grant information you provided in the ‘Funding Information’ and ‘Financial Disclosure’ sections do not match.

When you resubmit, please ensure that you provide the correct grant numbers for the awards you received for your study in the ‘Funding Information’ section.

We apologise for the disparity in the information provided in the ‘Funding Information’ and ‘Financial Disclosure’ sections. We have included a full financial disclosure in the manuscript which now correlates with the ‘Funding Information’ found within the editorial manager.

4. In your Data Availability statement, you have not specified where the minimal data set underlying the results described in your manuscript can be found. PLOS defines a study's minimal data set as the underlying data used to reach the conclusions drawn in the manuscript and any additional data required to replicate the reported study findings in their entirety. All PLOS journals require that the minimal data set be made fully available. For more information about our data policy, please see <http://journals.plos.org/plosone/s/data-availability>.

Upon re-submitting your revised manuscript, please upload your study’s minimal underlying data set as either Supporting Information files or to a stable, public repository and include the relevant URLs, DOIs, or accession numbers within your revised cover letter. For a list of acceptable repositories, please see <http://journals.plos.org/plosone/s/data-availability#loc-recommended-repositories>. Any potentially identifying patient information must be fully anonymized.

Important: If there are ethical or legal restrictions to sharing your data publicly, please explain these restrictions in detail. Please see our guidelines for more information on what we consider unacceptable restrictions to publicly sharing data: <http://journals.plos.org/plosone/s/data-availability#loc-unacceptable-data-access-restrictions>. Note that it is not acceptable for the authors to be the sole named individuals responsible for ensuring data access.

We will update your Data Availability statement to reflect the information you provide in your cover letter.

We have provided a more detailed data availability statement in the editorial manager. Hopefully this is more helpful but please do let us know if you require further information.

5. Please note that in order to use the direct billing option the corresponding author must be affiliated with the chosen institute. Please either amend your manuscript to change the affiliation or corresponding author, or email us at [plosone@plos.org](mailto:plosone@plos.org) with a request to remove this option.

Thank you for this guidance. We have contacted [plosone@plos.org](mailto:plosone@plos.org) to request that this option is now removed.

6. Please include your full ethics statement in the ‘Methods’ section of your manuscript file. In your statement, please include the full name of the IRB or ethics committee who approved or waived your study, as well as whether or not you obtained informed written or verbal consent. If consent was waived for your study, please include this information in your statement as well.

We have specified this information in full under the ‘Ethics’ heading, found within the ‘Methods’ section of the manuscript.

[Note: HTML markup is below. Please do not edit.]

Reviewers' comments:

Reviewer's Responses to Questions

**Comments to the Author**

1. Is the manuscript technically sound, and do the data support the conclusions?

The manuscript must describe a technically sound piece of scientific research with data that supports the conclusions. Experiments must have been conducted rigorously, with appropriate controls, replication, and sample sizes. The conclusions must be drawn appropriately based on the data presented.

Reviewer #1: Yes

Reviewer #2: Yes

2. Has the statistical analysis been performed appropriately and rigorously?

Reviewer #1: Yes

Reviewer #2: Yes

3. Have the authors made all data underlying the findings in their manuscript fully available?

The [PLOS Data policy](http://www.plosone.org/static/policies.action#sharing) requires authors to make all data underlying the findings described in their manuscript fully available without restriction, with rare exception (please refer to the Data Availability Statement in the manuscript PDF file). The data should be provided as part of the manuscript or its supporting information, or deposited to a public repository. For example, in addition to summary statistics, the data points behind means, medians and variance measures should be available. If there are restrictions on publicly sharing data—e.g. participant privacy or use of data from a third party—those must be specified.

Reviewer #1: Yes

Reviewer #2: Yes

4. Is the manuscript presented in an intelligible fashion and written in standard English?

PLOS ONE does not copyedit accepted manuscripts, so the language in submitted articles must be clear, correct, and unambiguous. Any typographical or grammatical errors should be corrected at revision, so please note any specific errors here.

Reviewer #1: Yes

Reviewer #2: Yes

5. Review Comments to the Author

Please use the space provided to explain your answers to the questions above. You may also include additional comments for the author, including concerns about dual publication, research ethics, or publication ethics. (Please upload your review as an attachment if it exceeds 20,000 characters)

Reviewer #1: Thank you for inviting me to review this comprehensive and interesting piece of work. Covid-19 has specifically devastated global well-being and all walks of life in 2020 and 2021. In this regard, this study has an essential and conducted critical study design. However, I want to add some sticky notes that will enhance the work's acceptability and quality and must be addressed before recommending it for publication.

1) A term used in abstract IMD, what is it? Be careful using abbreviations in the abstract and the main body of the paper before using their full form.

Many thanks for identifying this error. We have corrected this, spelling out the abbreviated term in full. Furthermore, we have ensured the remainder of the abstract and main body of the paper has abbreviations spelled out in full before using their abbreviated terms.

2) Please elaborate on the rationale behind using the ethnic code and their selection. Why did authors make a separate code for non-white British, Pakistani heritage, and others? Why didn't they consider the other ethnicity separately? Is it a focused study on Pakistani heritage mothers?

Thank you for this feedback. We have provided greater clarity behind the rationale for using these ethnic codes and why ethnicity was not considered separately in the manuscript.

3) Literature on stringent lockdown measures must also be added and compared with the UK government because the survey was conducted during this phase. In this regard, a couple of studies suggested.
10.1016/j.heliyon.2021.e05912
10.5055/jem.0529
10.5055/jem.0621
10.1080/00223980.2022.2039891

Thank you for your feedback and for the literature recommendations. Despite the Bradford population containing many mothers of Pakistani Heritage, we feel that the geographical and political setting of the research being limited to the UK government means that a comparative analysis of lockdown measures is perhaps not relevant to the findings of this paper at this time. However, we have included further references to literature exploring the scope and impact of the lockdown measures on this population in the UK.

4) The discussion section needs further work. Add relevant previous studies and compare their findings with your study results to show the study gaps and innovative points more clearly.

Thank you for this feedback. We have hopefully addressed this point with further reflection upon the results and comparison to other study results, as recommended by both reviewers.

5) Implication of the study findings and conclusions also needs more convincing arguments related to their practical implications and highlighting the innovative points of the study for policymakers.

We have also addressed this in the manuscript based on the recommendations.

Reviewer #2: Dear authors,

Kindly find below few comments to be considered:

1- In the abstract, add clearly the aim of the study, methodology, and recommendations.

Thank you for this feedback. We have amended the abstract to clearly add the study, methodology and recommendations.

2- After the introduction, you have to add "Literature review".

We have more clearly defined the literature review, with the inclusion of the ‘Literature Review’ heading within the introduction.

3- Since your used Survey to emphasize the research hypotheses or questions. In your study i did not see clear research hypotheses or questions. Authors should add Literature review and at the end of the literature review you should generate research hypotheses or questions. Then in the results section you should show us how your results confirm or refute the hypotheses or questions of the study.

Thank you for the feedback. We have reworked the introduction to improve the structure and signposting of the introduction. We have also reworked sections of the introduction to more clearly explain the aim and research questions of the study.

4- In the discussion section, you can laso show how the other studies support your results.

Thank you for this feedback. We have hopefully addressed this point with further reflection upon the results and comparison to other study results, as recommended by both reviewers.

5- In the results section, you should also clarify statistically how you can enphasize the hypotheses or questions and which statistics support each one.

Thank you for your feedback, we have amended the results section headings to make this clearer.

6- Usually the survey statistics should answer a research questions or hypotheses. I cannot see any of this relations.

Thank you for your feedback. Hopefully the amendments we have made along the lines of the feedback above have addressed this point to provide greater clarity regarding the research questions and how the research answers these questions.

Thank you once again for your consideration of this manuscript. If you require any further information please do let us know.

Yours sincerely,

Sian Reece
