# Supplementary material for: The long-term impact of the Covid-19 pandemic on financial insecurity in vulnerable families: Findings from the Born in Bradford Covid-19 longitudinal study
Source: PLoS One. 2023 Nov 29;18(11):e0295064. doi: 10.1371/journal.pone.0295064 (PMC10686492; doi:10.1371/journal.pone.0295064)
Supplement: Attachment — Submitted filename: Response to Reviewers.docx [file pone.0295064.s002.docx]

Sian Reece

Hull York Medical School

Heslington Road

York

YO10 5DD

1^st^ November 2023

Dear Wajiha Haq,

**Re: Manuscript PONE-D-23-05276 Revision Required**

Thank you for taking the time to consider our manuscript entitled “The long-term impact of the Covid-19 pandemic on financial insecurity in vulnerable families: findings from the Born in Bradford Covid-19 longitudinal study.”

We have carefully worked through the thoughtful comments of the editorial and reviewer teams and have provided our response to these comments for your consideration below.

Reviewer's Responses to Questions

**Comments to the Author**

1. If the authors have adequately addressed your comments raised in a previous round of review and you feel that this manuscript is now acceptable for publication, you may indicate that here to bypass the “Comments to the Author” section, enter your conflict of interest statement in the “Confidential to Editor” section, and submit your "Accept" recommendation.

2. Is the manuscript technically sound, and do the data support the conclusions?

The manuscript must describe a technically sound piece of scientific research with data that supports the conclusions. Experiments must have been conducted rigorously, with appropriate controls, replication, and sample sizes. The conclusions must be drawn appropriately based on the data presented.

Reviewer #1: Yes

3. Has the statistical analysis been performed appropriately and rigorously?

Reviewer #1: Yes

4. Have the authors made all data underlying the findings in their manuscript fully available?

The [PLOS Data policy](http://www.plosone.org/static/policies.action#sharing) requires authors to make all data underlying the findings described in their manuscript fully available without restriction, with rare exception (please refer to the Data Availability Statement in the manuscript PDF file). The data should be provided as part of the manuscript or its supporting information, or deposited to a public repository. For example, in addition to summary statistics, the data points behind means, medians and variance measures should be available. If there are restrictions on publicly sharing data—e.g. participant privacy or use of data from a third party—those must be specified.

Reviewer #1: Yes

5. Is the manuscript presented in an intelligible fashion and written in standard English?

PLOS ONE does not copyedit accepted manuscripts, so the language in submitted articles must be clear, correct, and unambiguous. Any typographical or grammatical errors should be corrected at revision, so please note any specific errors here.

Reviewer #1: Yes

6. Review Comments to the Author

Please use the space provided to explain your answers to the questions above. You may also include additional comments for the author, including concerns about dual publication, research ethics, or publication ethics. (Please upload your review as an attachment if it exceeds 20,000 characters)

Reviewer #1: As I Recommended in the previous review, please add some stringency measures in other countries or regions and compare them with your findings, which have not been considered. The purpose behind suggesting such measures is to compare what others have done and how the UK government would have done it better or effectively. If they also do the same standard measures, what is the difference, and how would you say your findings are innovative or novel?

There is no need to summarize the key findings in the discussion section. It would be a repetition of the summary with a conclusion section. Just mention this summary while concluding remarks.

Thank you for your feedback. We have addressed these points in the manuscript.

7. PLOS authors have the option to publish the peer review history of their article ([what does this mean?](https://journals.plos.org/plosone/s/editorial-and-peer-review-process#loc-peer-review-history)). If published, this will include your full peer review and any attached files.


If you choose “no”, your identity will remain anonymous but your review may still be made public.


**Do you want your identity to be public for this peer review?** For information about this choice, including consent withdrawal, please see our [Privacy Policy](https://www.plos.org/privacy-policy).

Reviewer #1: No

Thank you once again for your consideration of this manuscript. If you require any further information please do let us know.

Yours sincerely,

Sian Reece
